# Aquaporins in fishes—expression, localization, and functional dynamics

**DOI:** 10.3389/fphys.2012.00434

**Published:** 2012-11-12

**Authors:** Steffen S. Madsen

**Affiliations:** Institute of Biology, University of Southern DenmarkOdense, Denmark

By living in a “world of water” fishes are exposed to major osmotic challenges that are opposite in nature in the freshwater and marine environments. In both cases, obligatory water fluxes primarily due to osmotic gradients across respiratory surfaces are threatening to the internal milieu and must be compensated by bulk flows of water in the opposite direction. While the ionoregulatory mechanisms that generate the osmotic driving force for such water flows have been known for decades, the molecular pathways of compensatory water fluxes are still largely unravelled. Current models suggest that water passes hydrophobic epithelia by para- and/or transcellular pathways, the former being defined by the characteristics of tight junctions, the latter determined by the serial permeability of apical and basolateral cellular membranes. Transcellular water transport may occur by simple diffusion through lipid bilayers or become markedly improved by the insertion of integral channel proteins (aquaporins) in the plasma membrane. Thus, aquaporins can truly be conceived as the plumbing system of cells.

In mammals, 13 aquaporin subfamilies are present and several of these have been investigated structurally and functionally in >6600 publications since their discovery in 1992 by Agre and colleagues. The first paper on aquaporins in fishes appeared in Cutler and Cramb (2000) but surprisingly few papers have addressed aquaporins in fishes and other non-mammalian vertebrates during the ensuing decade. More recently, it has been established that the zebrafish and other teleosts retain up to 18 aquaporin genes with homologies to all but a few of the mammalian orthologues. However, the forthcoming publishing of the Atlantic salmon genome may add even more paralogues to this list (Finn, pers. communication). One of the major challenges ahead of us is therefore to describe and understand the differentiated functionality of such diversity.

Aquaporins most certainly play distinct roles in fishes as they do in mammals—both at the cellular, organ and organismal level. However, there is a considerable lack of information from the fish world on this topic, with only ca. 50 papers addressing aquaporins in fishes at the time of the call of this Research Topic in 2010. Most of these describe tissue expression patterns in various Teleosts, while Aqnathans and Chondrichthyes and the functionality of fish aquaporins have received very little focus. This ebook presents a collection of papers addressing the evolution and role of aquaporins during gametogenesis and embryonic development as well as for water transport across adult gill, kidney and intestinal epithelia using bony and cartilagenous fishes as models. Our aim is to stimulate new original research in this area as well as bringing together new collaborations across fields.
